# Inhibition of EZH2 induces NK cell-mediated differentiation and death in muscle-invasive bladder cancer

**DOI:** 10.1038/s41418-019-0278-9

**Published:** 2019-01-28

**Authors:** Swathi Ramakrishnan, Victoria Granger, Monika Rak, Qiang Hu, Kristopher Attwood, Lanni Aquila, Nithya Krishnan, Rafal Osiecki, Gissou Azabdaftari, Khurshid Guru, Gurkamal Chatta, Geraldine Gueron, Lacey McNally, Joyce Ohm, Jianmin Wang, Anna Woloszynska

**Affiliations:** 1 Department of Pharmacology and Therapeutics, Roswell Park Comprehensive Cancer Center, Buffalo, NY 14263 USA; 2 Department of Cell Biology, Jagiellonian University, 31-007 Krakow, Poland; 3 Department of Bioinformatics and BioStatistics, Roswell Park Comprehensive Cancer Center, Buffalo, NY 14263 USA; 4 Child Jesus Hospital, 02-005 Warsaw, Poland; 5 Department of Pathology, Roswell Park Comprehensive Cancer Center, Buffalo, NY 14263 USA; 6 Department of Urology, Roswell Park Comprehensive Cancer Center, Buffalo, NY 14263 USA; 7 Department of Medicine-GU Center, Roswell Park Comprehensive Cancer Center, Buffalo, NY 14263 USA; 8 Department of Biological Chemistry, University of Buenos Aires, IQUIBICEN-CONICET, Intendente Guiraldes 2160, CABA, 1428 Buenos Aires, Argentina; 9 Department of Cancer Biology, Wake Forest Comprehensive Cancer Center, Winston-Salem, NC 27157 USA; 10 Department of Cancer Genetics and Genomics, Comprehensive Cancer Center, Buffalo, NY 14263 USA

## Abstract

Lysine-specific demethylase 6A (KDM6A) and members of the Switch/Sucrose Non-Fermentable (SWI/SNF) family are known to counteract the activity of Enhancer of Zeste Homolog 2 (EZH2), which is often overexpressed and is associated with poor prognosis in muscle-invasive bladder cancer. Here we provide evidence that alterations in chromatin modifying enzymes, including KDM6A and members of the SWI/SNF complex, are frequent in muscle-invasive bladder cancer. We exploit the loss of function mutations in KDM6A and SWI/SNF complex to make bladder cancer cells susceptible to EZH2-based epigenetic therapy that activates an immune response to drive tumor cell differentiation and death. We reveal a novel mechanism of action of EZH2 inhibition, alone and in combination with cisplatin, which induces immune signaling with the largest changes observed in interferon gamma (IFN-γ). This upregulation is a result of activated natural killer (NK) signaling as demonstrated by the increase in NK cell-associated genes *MIP-1α, ICAM1, ICAM2*, and *CD86* in xenografts treated with EZH2 inhibitors. Conversely, EZH2 inhibition results in decreased expression of pluripotency markers, ALDH2 and CK5, and increased cell death. Our results reveal a novel sensitivity of muscle-invasive bladder cancer cells with KMD6A and SWI/SNF mutations to EZH2 inhibition alone and in combination with cisplatin. This sensitivity is mediated through increased NK cell-related signaling resulting in tumor cell differentiation and cell death.

## Introduction

The tumor suppressor Switch/Sucrose Non-Fermentable (SWI/SNF) complex [1–3] and Polycomb Repressive Complex (PRC2), of which the oncogene Enhancer of Zeste Homologue 2 (EZH2) [4–6] is the catalytic component, have opposing roles in regulation of gene transcription [7]. SWI/SNF family members displace PRC2 on target gene loci to allow gene transcription [8, 9]. Malignant rhabdoid and ovarian tumors with SWI/SNF family member mutations are believed to be dependent on EZH2 activity and thus more sensitive to EZH2 inhibition [10**–**15]. EZH2 function is also antagonized by Lysine-specific Demethylase 6A (KDM6A) to activate gene transcription of E-cadherin, cell cycle regulators, tumor suppressor STF amongst others [16**–**18]. KDM6A removes trimethylation marks from histone 3 lysine 27 (H3K27) [19] and its catalytic JmjC domain is essential for histone demethylase function [20, 21].

Similar to rhabdoid and ovarian tumors with SWI/SNF mutations [10**–**15], complete loss of KDM6A protein sensitizes bladder cancer cell lines and patient-derived xenografts to EZH2 inhibition [22]. EZH2 sensitivity is attributed to IGFBP3 upregulation in KDM6A-null cells, but not in wild-type KDM6A cells [22]. This EZH2 sensitivity in bladder cancer is based on total loss of KDM6A protein. In muscle-invasive bladder cancer (MIBC), KDM6A and members of the SWI/SNF family members are frequently mutated [23, 24], while EZH2 is overexpressed in tumors compared to adjacent non-tumor areas [25, 26]. EZH2 inhibition in the context of SWI/SNF family member and/or KDM6A mutations, but not necessarily at protein level alterations, in MIBC is unexplored.

Here we show that EZH2 inhibition is most effective in bladder cancer cells with both SWI/SNF family member and KDM6A mutations, and is capable of augmenting cisplatin response. We show for the first time that EZH2 inhibition in HT1376 xenografts with KDM6A and SWI/SNF family member mutations activates a natural killer (NK) cell-based immune response. NK cell activity was detected by *IFN-γ* upregulation and increased protein levels of Neural Cell Adhesion Marker (NCAM/CD56) and Natural Cytotoxicity triggering Receptor 1 (NCR1). Our results indicate that EZH2 inhibition alone and in combination with cisplatin boosts NK cell response to drive tumor differentiation and death in bladder cancer cells and xenografts. Therefore, we conclude that epigenetic therapy targeting EZH2 alone or in combination with cisplatin can be beneficial in bladder tumors with KDM6A and/or SWI/SNF mutations and/or increased EZH2 activity.

## Materials and methods

### Roswell Park Comprehensive Cancer Center (Roswell Park) patient cohort

Tumor samples from patients with MIBC and with informed consent were collected at the time of radical cystectomy at Roswell Park. RNA and exome sequencing of de-identified tumors were conducted.

### Cell culture

HT1376, T24, and UM-UC-3 cells were obtained from ATCC, and cultured in MEM, McCoy’s, and DMEM media, respectively, supplemented with 10% fetal bovine serum, and penicillin/streptomycin. Overall, 10 mM EPZ011989 stock solution was thawed no more than four times from −20 °C and diluted in media for treating cells at 1 µM concentration. In vitro treatments lasted 13 days. Initial treatment of cells with EPZ011989 occurred on days 1 and 4. Cells were harvested and re-plated at day 7 followed by additional EPZ011989 treatment on day 8. 1.0 mg/mL cisplatin was diluted to 0.25 µg/mL in media for treatment on day 11. On day 13, cells were harvested for western blots, clonogenic, and cell cycle assays. For siEZH2 experiments, cells were treated with 50 nM siRNA (Dharmacon, L-004218-00-0005) for 96 h.

### Western blots

Cells were trypsinized for histone extraction as per the Abcam protocol. Additionally, cells were lysed using RIPA buffer for whole-cell lysates. Protein concentration was assessed (BioRad, 5000116). A total of 10 µg total histones and 40 µg whole-cell lysates were loaded on gels. Membranes were incubated overnight at 4 °C with primary antibody in 5% BSA in TBST. Primary antibodies used were: H3K27me3 (Cell signaling, 9733S), total histone H3 (Cell Signaling, 9715), EZH2 (Cell signaling, 5246), KDM6A (Atlas Antibodies, HPA002111), ALDH2 (Abcam, ab108306), and CK5 (Covance, PRB 160-P). Membranes were incubated with HRP-conjugated secondary rabbit antibody (GE Life Sciences, NA934V). The secondary antibody was detected using Luminata Crescendo Western HRP Substrate (WBLUC0500). The membranes were stripped with Restore Plus (Thermo Scientific, 46428) and re-probed with GAPDH (Abcam, ab9485) or Total H3.

### Clonogenic assay

50 (T24), 100 (UM-UC-3), and 200 (HT1376) cells were plated onto 6-well plates to form clones for 9–12 days. Plates were rinsed with PBS, stained with crystal violet for 10 mins, and rinsed again. Overnight dried clones were counted.

### Cell cycle analysis

Cells were harvested with trypsin and re-suspended in 75% ethanol. Pellets were washed twice with PBS and re-suspended in 50 µg/mL propidium iodide (ThermoFisher, P3566) and 5 µg/mL ribonuclease (ThermoFisher, EN0531). Fortessa instrument and ModFit software were used for cell cycle analysis.

### In vivo study

The national guidelines for the use of laboratory animals were followed. 3 × 10^6^ HT1376 cells were injected subcutaneously in the left flank of nude mice. 100-250 mm^3^ tumors were treated with vehicle, cisplatin (3 mg/kg, I.P, weekly once), EPZ011989 (500 mg/kg, oral gavage, every 12 h) and combination therapy for 22 days. Body weights were measured before treatments for dosing and toxicity. Tumors were blindly measured twice per week. On day 22, mice were killed, tumors were processed for RNA-sequencing and immunohistochemistry (IHC).

### RNA-sequencing

Frozen tumors were crushed, and RNAs were isolated (ZymoResearch, R2052). Paired-end RNA-sequencing was performed on end-point tumors with fastQC assessing the base quality of raw reads. Tophat 2 was used to align reads to human genome b37 from Ensembl. Raw read counts were analyzed using R, and differentially expressed genes between four groups were identified. *Z*-scores were calculated for PRC2, stem cell-like and NK cell signatures. Boxplots were used to show the *z*-scores for each signature in each sample set. Wilcoxon-paired test were used to test the difference among the three signatures in each sample set. Log CPM (count per million) were used to quantify the expression of each gene and then normalized by the number of standard deviations from the mean. The sum of the normalized values of signature genes was calculated as the *z*-score for each sample.

### RNA-sequencing validation

cDNA was prepared (BioRad, 170-8891). SYBR green (BioRad, 172-5121) was used for qPCR with primers listed below:

### Immunohistochemistry

Tumors were fixed in 10% Neutral Buffer Formalin (Sigma, HT-501126), paraffin embedded and stained with hematoxylin and eosin, EZH2, H3K27me3, Ki67 (Abcam, ab15580), ALDH2, CK5, p63, CD56 (Cell signaling, 3576), and TUNEL (R&D, 4828-30-DK). Three scorers blindly scored H3K27me3. All the other staining’s were analyzed by Image J to calculate the area fraction for CK5, CD56 and NCR1 (Abcam, ab214468) as well as percent positive nuclei (by ImmunoRatio [27]) for Ki67, p63, and EZH2. The staining’s were also subjected to blinded observation by the pathologist.

### Statistical analysis

GraphPad Prism was used for graphs and statistical analysis. In vitro experiments were compared by student’s *t*-test. For mathematical modeling, tumor volumes by treatment and time point were reported using mean and standard deviation and graphically represented by mean plots (mean ± standard error). Missing tumor volumes were extrapolated using linear regression based on a given mouse’s observed log-tumor volumes. Linear mixed was used to model log (volume) as a function of treatment, time, their interaction, and random mouse effect. The fitted model estimated how long it would take a tumor at 100 mm^3^ to reach a volume of 1000 mm^3^. The association between last observed tumor volumes and treatment groups were evaluated using one-way ANOVA model; with pairwise comparisons made as appropriate. H3K27me3 scores were calculated as: total score = 1*(# cells scored 1) + 2*(# cells scored 2) + 3*(# cells scored 3). The resulting H3K27me3 scores ranged from 0 (all cells scored as 0) to 600 (all cells scored as 3). H3K27me3 scores were modeled as a function of treatment and random sample effect using a linear mixed model with assumptions verified graphically. Mean H3K27me3 scores were compared between groups using Tukey-adjusted tests about the least square means. Time-to-event outcomes were reported by EZH2/KDM6A status using standard Kaplan–Meier methods; with log-rank test comparisons. All analyses were conducted in SAS v9.4 (Cary, NC) at a significance level of 0.05.

## Results

### Mutations and altered expression of chromatin remodeling genes are recurrent in MIBC patients

**Fig. 1:**
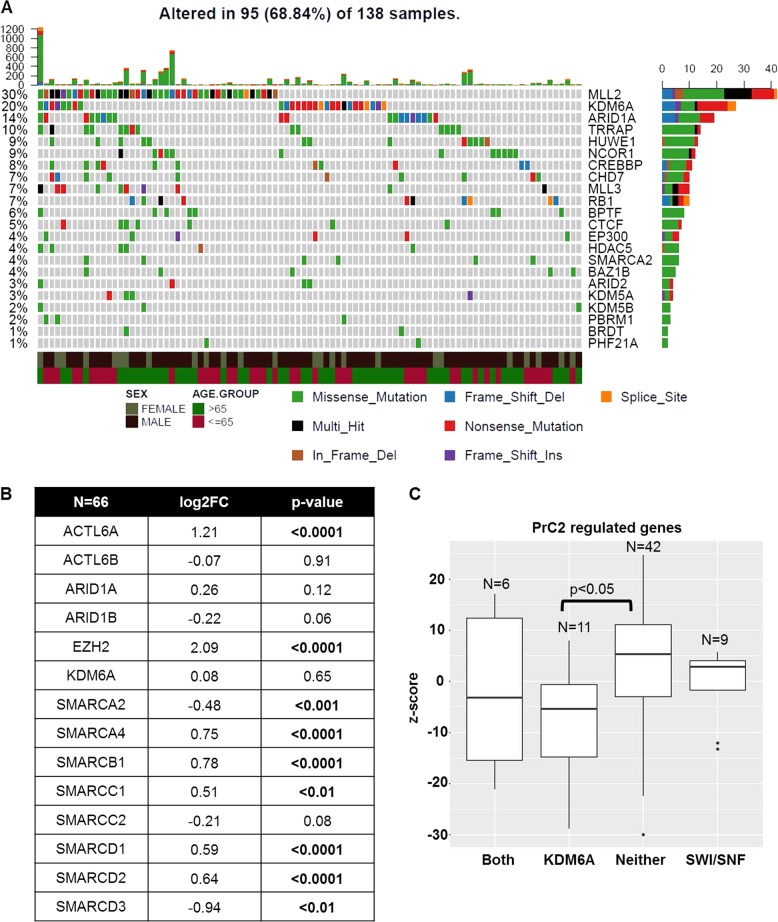
Mutations in KDM6A and SWI/SNF complex genes are common in muscle-invasive bladder cancer. **a** Exome-sequencing analysis of 138 bladder tumor patients from Roswell Park Comprehensive Cancer Center (Roswell Park) revealed mutations in several chromatin remodeling enzymes. KMD6A, a histone demethylase, responsible for removing trimethylation mark on H3K27 is mutated in 20% while SWI/SNF complex family members are mutated in 18% of tumors. Mutations include missense and nonsense mutations as well as frameshift deletions, insertions and in frame deletions. **b** RNA-sequencing of a 66 tumors revealed significant alterations in gene expression of *EZH2* and members of the SWI/SNF complex. **c** *z*-scores of PRC2-regulated genes were lower in tumors with KDM6A and KDM6A/SWI/SNF mutations compared to tumors without any mutations or SWI/SNF mutations alone

Neoadjuvant cisplatin-based chemotherapy followed by either radical cystectomy or PD-L1-based immunotherapy are the only two options for patients with MIBC that has spread locally or regionally. Cisplatin-treated patients often recur while PD-L1-based therapy is only effective in 20% of the patients [28**–**30]. It is therefore critical not only to identify therapies effective in patients with cisplatin failure but also increase the probability of targeted therapy success based on the molecular characteristics of patient tumors. For this purpose, we systematically analyzed bladder tumor tissues from patients treated at Roswell Park by exome and RNA-sequencing. 20% of Roswell Park tumors exhibited frameshift, nonsense and missense mutations in Lysine-specific Demethylase 6A (KDM6A), that specifically removes methylation marks from histone 3 lysine 27 (H3K27) (Fig. 1a, Supplementary Table 1). SWI/SNF complex family members that regulate transcription by chromatin remodeling [31], including ACTL6B, ARID1A, and SMARCA4, were mutated in 18% of tumors (Fig. 1a, Supplementary Table 1). 10% of tumors had concurrent mutations in both KDM6A and the SWI/SNF family members. These frequencies of mutations were similar to The Cancer Genome Atlas (TCGA) bladder cancer cohort (Supplementary Figure 1A).

RNA-sequencing also revealed that a subset of SWI/SNF family members and *EZH2* transcripts were significantly altered in tumors compared to non-tumor tissues (Fig. 1b). We further categorized muscle-invasive tumors based on their mutational status as KDM6A alone, SWI/SNF alone, both KDM6A and SWI/SNF and neither mutation. We analyzed genes that are repressed by Polycomb Repressive Complex 2 [32], of which EZH2 is the catalytic subunit, in these mutational subtypes. Tumors with KDM6A only and KDM6A/SWI/SNF family member mutations had lower *z*-scores of PRC2 targets compared to tumors with neither mutation (Fig. 1c). This suggests that tumors with KDM6A only and KDM6A/SWI/SNF mutations have increased EZH2 activity and can potentially be sensitive to EZH2 targeted therapy. In TCGA, patients with either loss of function mutations or mRNA downregulation in either KDM6A or SWI/SNF family members had worse overall (30.91 vs. 61.4 months) and progression-free survival (17.35 vs. 87.48 months, *p* < 0.05) compared to patients with no alterations (Supplementary Figure 1B). Patients with EZH2 amplification and/or mRNA overexpression had shorter overall (28.38 vs. 33.11 months) and progression-free survival (27.83 vs. 32.59 months) compared to patients with no alterations (Supplementary Figure 1B). We did not have adequate patient numbers in the Roswell Park cohort to perform survival analysis. In summary, common genetic and transcriptome level alterations in chromatin remodeling genes in muscle-invasive bladder tumors can provide a framework that could be exploited for novel epigenetic-based therapy. Combined, these data indicate that EZH2 inhibition could be a rationale therapeutic strategy for a significant subset of MIBC.

### Cells with both KDM6A and SWI/SNF mutations are sensitive to EZH2 inhibition

**Fig. 2:**
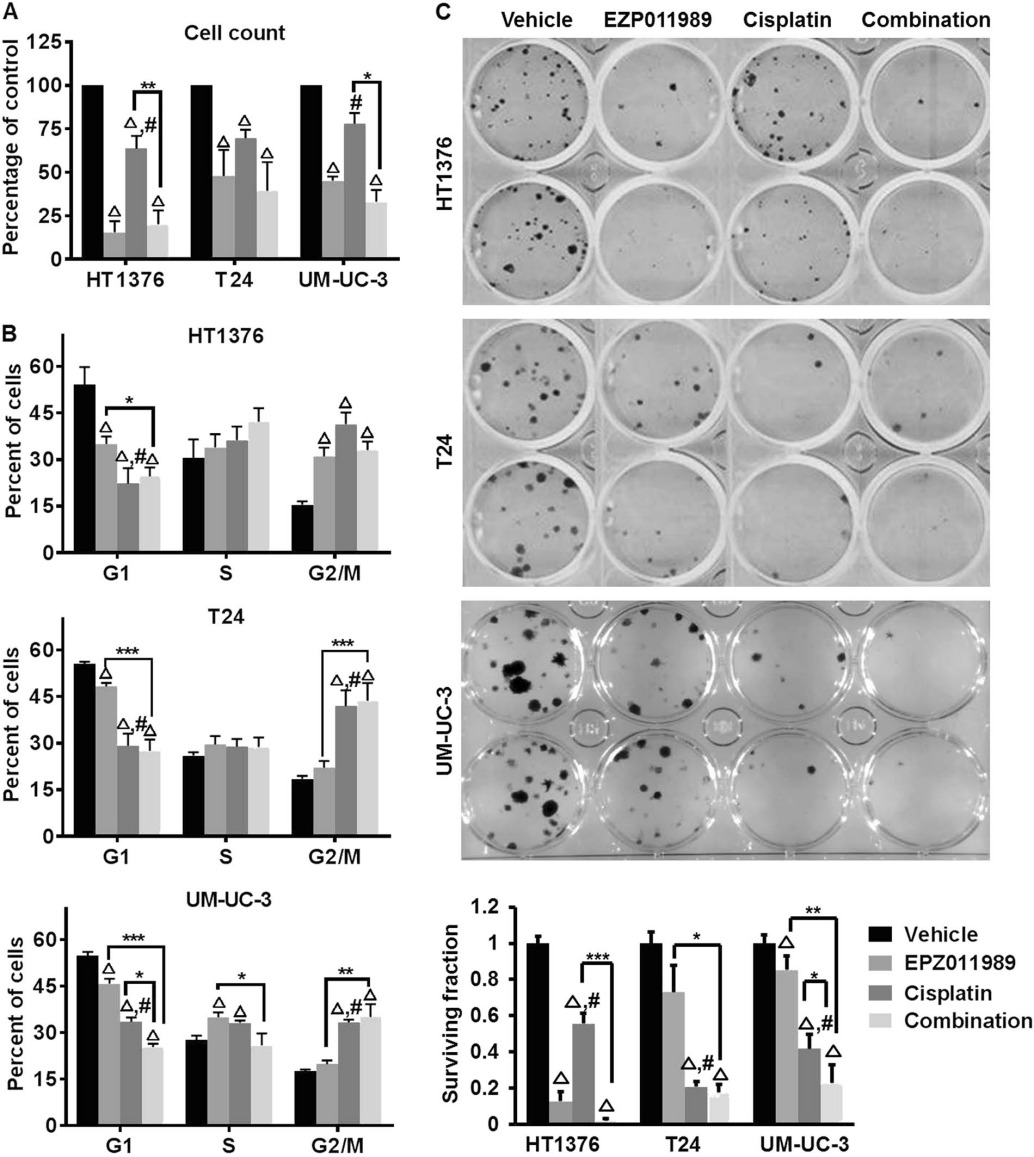
Bladder cancer cells with ARID1A and/or SWI-SNF mutations are sensitive to EZH2 inhibition. **a** 1 µM EPZ011989 alone or in combination with 0.25 µg/ml cisplatin reduced the number of viable cells compared to vehicle. The *Y*-axis represents the percentage of cells normalized to the vehicle, and the *X*-axis represents the cell lines used in the study. **b** EPZ011989 alone or in combination with cisplatin increased the number of cells in the G2/M phase of the cell cycle. The *Y*-axis represents the percentage of propidium iodide positive cells, and the *X*-axis represents the different phases of the cell cycle. **c** The image is a representative of clonogenic assay performed on all three cell lines. EPZ011989 alone or in combination with cisplatin significantly reduced the number of clones. The *Y*-axis represents the surviving fraction of clones normalized to the vehicle. Error bars represent standard error of means from technical duplicates and biological triplicates. Students *t*-test was used to analyze significance between the single agent and combination treatments. *p < 0.05, **p < 0.01, ***p < 0.001, triangle indicates significant difference compared to vehicle, hash indicates a significant difference between the EPZ011989 and cisplatin alone treatments

The SWI/SNF complex and KDM6A oppose EZH2-mediated transcriptional repression [7, 19]. Tumors with either SWI/SNF or KDM6A mutations are thought to be sensitive to EZH2 inhibition [13, 22, 33]. Therefore, we investigated whether EZH2 inhibition differentially affects bladder cancer cell lines with mutations in either KDM6A alone or both KDM6A and SWI/SNF family members. In our studies, we used: (1) HT1376 (KDM6A and SWI/SNF family mutations), (2) T24 (KDM6A nonsense mutation) and, (3) UM-UC-3 (KDM6A and SMARCA4 mutations) (Supplementary Table 2 [34]). First, we confirmed the activity of the EZH2 inhibitor, EPZ011989, by testing H3K27me3 protein levels after 4 days of treatment. H3K27me3 levels were reduced to similar levels in HT1376 and UM-UC-3 cells treated with 0.01 µM EPZ011989 as T24 cells treated with 1 µM EPZ011989 (Supplementary Figure 2A). Therefore, we used 1 µM EPZ011989 to ensure H3K27me3 reduction in all three cell lines. We also tested H3K27me3 levels in short-term cisplatin-treated cells, since cisplatin is the first-line therapy for MIBC patients. 0.25 µg/mL of cisplatin, a sub-toxic cisplatin concentration [35], increased H3K27me3 levels in all three cell lines (Supplementary Figure 2B). Cisplatin-mediated H3K27me3 alterations prompted us to test the effects of EPZ011989 alone and in combination with cisplatin on cell proliferation, cell cycle and clonogenicity in vitro. EPZ011989 alone and in combination with cisplatin significantly reduced cell proliferation, caused G2/M arrest and reduced clonogenicity of HT1376 cells (Fig. 2a–c, Supplementary Table 3). In T24 and UM-UC-3 cells, EPZ011989 alone and in combination with cisplatin inhibited cell proliferation, but only the combination caused significant G2/M arrest and reduced clonogenicity compared to vehicle and single agent treatments (Fig. 2a–c, Supplementary Table 3). The G2/M arrest in cisplatin-treated cells did not correspond to reduced cell proliferation (Fig. 2a, b). It is worth noting that we used sub-toxic doses of cisplatin [35] that may not robustly reduce cell proliferation in these cells. T24 and UM-UC-3 cell lines were more sensitive to cisplatin in the clonogenic assay than the cell proliferation assay. We think that the cisplatin concentration used in our study reduces the long-term ability of cells to form individual clones, but does not affect short-term (48 h) proliferation. In summary, our in vitro assays suggest that HT1376 cells with both KDM6A and multiple SWI/SNF family member mutations are more sensitive to EZH2 inhibition alone compared to T24 and UM-UC-3 cells.

**Fig. 3:**
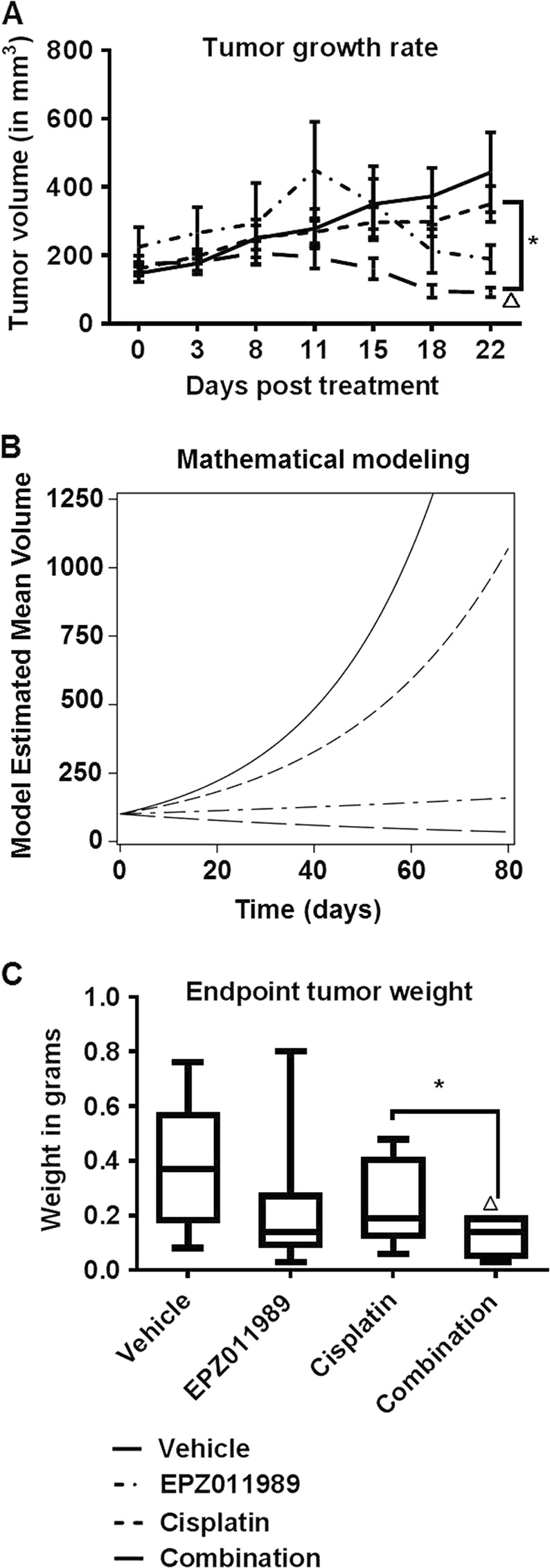
HT1376 xenografts with KDM6A and ARID1A mutations are sensitive to EZH2 inhibition alone and in combination with cisplatin. **a** 500 mg/kg EPZ011989 alone or in combination with 3 mg/ml cisplatin reduced HT1376 tumor volume in nude mice. The *Y*-axis represents tumor volume in mm^3^ and the *X*-axis represents number of days in the study. There is a significant difference in the tumor growth rate between the combination treatment and cisplatin alone treatment. **b** Mathematical modeling of tumor growth rate indicated that the vehicle, Cisplatin, EPZ011989 would grow tumors from 100 to 1000 mm^3^ in 58.5, 77.7, and 399.8 days, respectively. The combination treatment is predicted to not reach 1000 mm^3^. **c** End-point tumor weights showed that the combination treatment had significantly lower tumor weight as compared cisplatin alone treatment. The *Y*-axis represents the tumor weight in grams and the *X*-axis represents the different groups. Error bars represent standard error of means of all the mice in each group. One-way ANOVA model with pairwise comparisons made as appropriate were performed to test differences between single and combination treatments. *p < 0.05, **p < 0.01, ***p < 0.001, triangle indicates significant difference as compared to vehicle, hash indicates significant difference between the EPZ011989 and cisplatin alone treatments

**Fig. 4:**
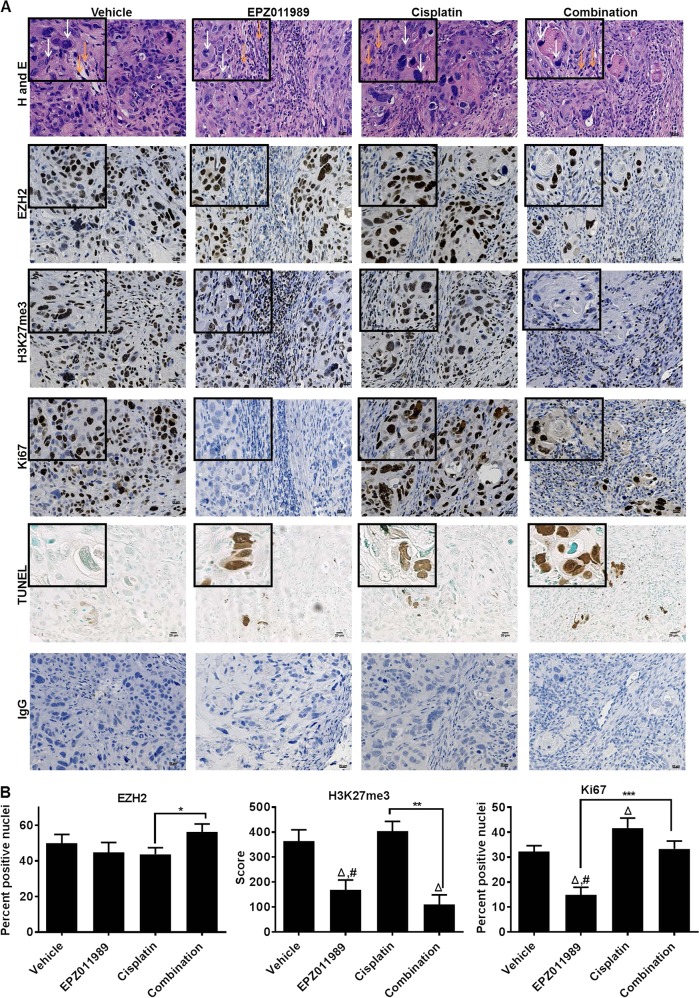
EZH2 inhibition alone and in combination with cisplatin reduces cell proliferation and increases cell death in HT1376 xenografts. **a**, **b** Representative H and E images showed no difference in tumor morphology between the different treatments. EZH2 expression was not altered between the treatment groups with a slight increase in combination-treated tumors; however, H3K27me3 levels were lower in the EPZ011989 and combination groups. Ki67, a proliferation marker, was reduced in EPZ011989 and combination groups compared to vehicle and cisplatin-treated tumors. TUNEL staining, an apoptosis marker was higher in all treatment groups compared with vehicle. Error bars represent standard error of means of all the mice in each group. One-way ANOVA model with pairwise comparisons made as appropriate were performed to test differences between single and combination treatments. *p < 0.05, **p < 0.01, ***p < 0.001, triangle indicates significant difference as compared to vehicle, hash indicates significant difference between the EPZ011989 and cisplatin alone treatments

Next, we used HT1376-derived xenografts to determine whether sensitivity to EZH2 inhibition in vitro is maintained in vivo. Similar to observations in vitro, EPZ011989 and combination treatments reduced HT1376 xenograft growth compared to vehicle and cisplatin-treated mice (Fig. 3a, Supplementary Table 4) without causing significant body weight loss (Supplementary Figure 3A–E). To determine the long-term effect on xenograft growth, we used mathematical modeling to calculate the rate of tumor growth to a predetermined end-point volume, 1000 mm^3^. The model predicted that xenografts from vehicle, cisplatin, and EPZ011989 would reach a volume of 1000 mm^3^ in 58.5, 77.7, and 399.8 days, respectively, but the combination would prevent xenografts from reaching 1000 mm^3^ (Fig. 3b). Additionally, EPZ011989 and combination-treated xenografts were predicted to shrink compared to vehicle and cisplatin groups. At the end of the study, EPZ011989 (0.258 + 0.054 g) and combination (0.123 ± 0.027 g)-treated xenografts were significantly smaller than vehicle (0.373 ± 0.093 g) and cisplatin (0.219 ± 0.079 g)-treated tumors (Fig. 3c, Supplementary Table 4). Correspondingly, EPZ011989 and combination-treated xenografts had reduced Ki67 staining, a cell proliferation marker, compared to vehicle and cisplatin-treated xenografts (Fig. 4a, b, Supplementary Table 4). To analyze whether reduced proliferation was due to increased cell death in treatment groups, we performed TUNEL staining, a marker of cell death, on tumor sections. We found greater TUNEL positivity in EPZ011989, cisplatin and combination-treated xenografts (Fig. 4a). Finally, we confirmed EPZ011989 activity in vivo by quantitating H3K27me3 staining by immunohistochemistry. H3K27me3 scores were significantly lower in EPZ011989 (168.5 ± 39.7) and combination (110 ± 37.7)-treated xenografts compared to vehicle (364.2 ± 45.0) and cisplatin (430.4 ± 39.7)-treated xenografts without obvious changes in EZH2 protein expression (Fig. 4a, b, Supplementary Table 4). In summary, HT1376-derived xenografts maintain sensitivity to EZH2 inhibition alone and in combination with cisplatin in vivo.

### Transcripts associated with NK cell activity are elevated with EZH2 inhibition

**Fig. 5:**
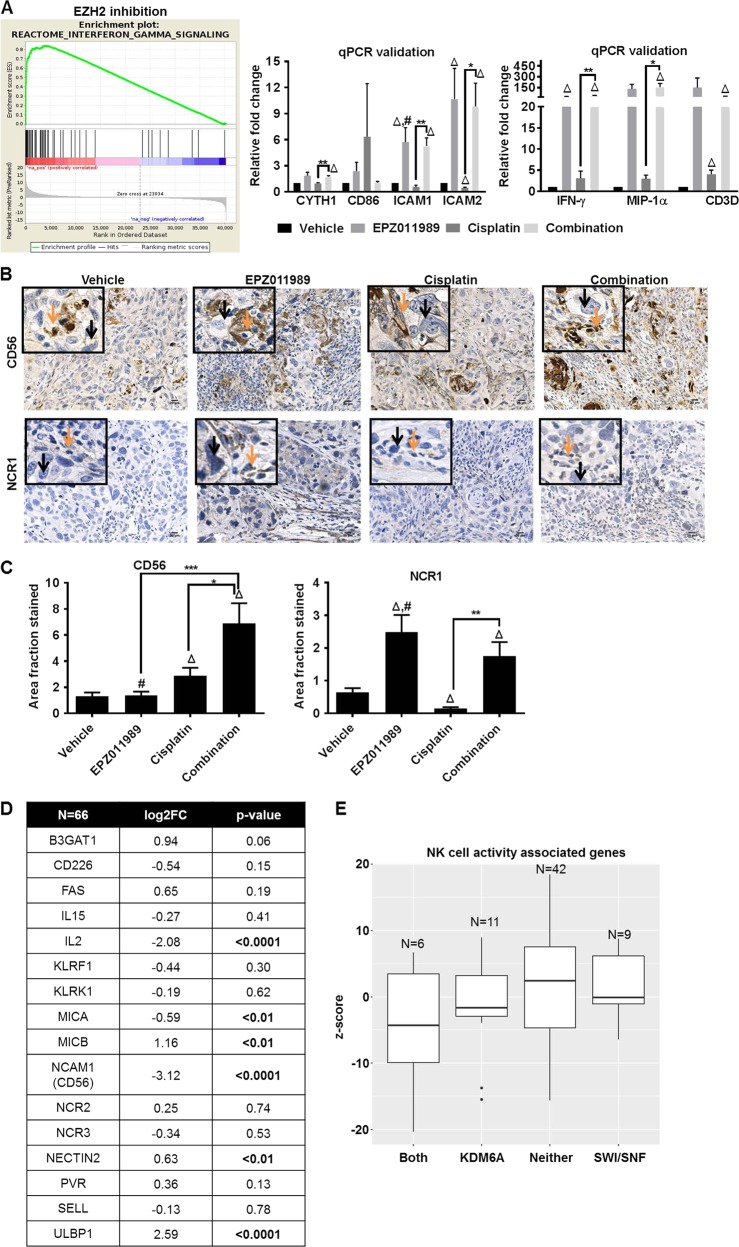
EZH2 inhibition is associated with NK cell activation. **a** Gene set enrichment analysis shows that EZH2 inhibition alone or in combination with cisplatin is associated with increased in IFN-γ signaling. qPCR analysis showed that NK cell activity associated transcripts *IFN-γ*, *MIP-1α*, *CD3D*, *CYTH1*, *CD86*, *ICAM1*, and *ICAM2* were significantly upregulated in the combination treatment as compared to cisplatin alone treatment. Error bars represent standard error of means of all the mice in each group. One-way ANOVA model with pairwise comparisons made as appropriate were performed to test differences between single and combination treatments. *p < 0.05, **p < 0.01, triangle indicates significant difference as compared to vehicle, hash indicates significant difference between the EPZ011989 and cisplatin alone treatments. **b**, **c** CD56, an NK cell-associated marker is significantly higher in combination-treated tumors compared to single agent and vehicle-treated mice. NCR1, Natural Cytotoxic triggering Receptor 1, that mediates tumor cell lysis is significantly increased in the EPZ011989 and combination-treated groups compared to vehicle and cisplatin-treated groups. Orange arrows indicate infiltrating cells and black arrows point to tumor cells. Error bars represent standard error of means of Image J quantitation in all the mice in each group. One-way ANOVA model with pairwise comparisons made as appropriate were performed to test differences between single and combination treatments. *p < 0.05, **p < 0.01, triangle indicates significant difference as compared to vehicle, hash indicates significant difference between the EPZ011989 and cisplatin alone treatments. **d** RNA-sequencing of a 66 tumors revealed significant alterations in gene expression of a subset of ligands and receptors that are involved in the activation of natural killer (NK) cells. **e** *z*-scores of NK related transcripts are higher in tumors with no mutations compared to tumors with KDM6A and/or SWI/SNF mutations

To understand the potential molecular mechanism of EZH2 inhibition-mediated anti-tumor activity in our studies, we used an unbiased RNA-sequencing approach. We performed RNA-sequencing on HT1376 and T24 cells treated for 4 days with EPZ011989 and HT1376 xenografts. We focused on upregulated transcripts as these are more likely to be the direct result of increased gene transcription due to decreased H3K27me3 by EZH2 inhibition. *IFN-γ* RNA transcripts had the highest fold change in EPZ011989 and combination-treated HT1376 xenografts (Supplementary Table 5). Transcripts associated with IFN-γ signaling were significantly enriched only in those xenografts treated with EPZ011989, and not in vehicle or cisplatin-treated mice (Fig. 5a). IFN-γ is one of the downstream effectors of activated NK cells, and it is important to note that athymic nude mice still have active NK, B, and dendritic cells. Using qPCR, we validated upregulation of a set of transcripts associated with NK cell activity, including *IFN-γ*, *CYTH1*, *CD86*, *ICAM1*, *ICAM2*, *MIP-1α*, and *CD3D* in EPZ011989 and combination-treated xenografts (Fig. 5a). Interestingly, we found a large number of immune associated transcripts in short-term EPZ011989-treated HT1376 and T24 cells in vitro (Supplementary Figure 4A–D, Supplementary Table 6–9). This suggests that EZH2 inhibition alters expression of immune signaling transcripts in tumor cells in vitro, and in vivo in presence of a limited immune microenvironment.

To evaluate whether transcript level changes in NK cell-associated signaling increased infiltrating lymphocytes in EPZ011989-treated xenografts, we used CD56 also known as Neural Cell Adhesion Molecule 1 (NCAM1) and NCR1 (Natural Cytotoxicity triggering Receptor 1) as markers of NK cells [36]. CD56 staining was significantly higher in combination-treated xenografts compared with vehicle, cisplatin and EPZ011989-treated mice (Fig. 5b, c, Supplementary Table 4). Similarly, NCR1 was significantly higher in EPZ011989 and combination-treated tumors compared to vehicle and cisplatin-treated groups (Fig. 5b, c, Supplementary Table 4). NK cells are known to mediate tumor cell lysis [37] and we observed association of increased NK cell infiltration with increased TUNEL staining in EZH2 inhibitor-treated xenografts. Overall, these results suggest that EPZ011989 alone and in combination with cisplatin can increase NK cell infiltration and thereby tumor cell cytotoxicity in HT1376 xenografts.

To determine whether NK cells have any MIBC clinical relevance, we examined transcript levels of NK cell ligands and receptors in patient tumors. NK cell-associated transcripts, both ligands and receptors, were consistently downregulated in the Roswell Park cohort (Fig. 5d) with the exception of ULBP1, an NK cell ligand. Although ULBP1 ligand is upregulated, the NKG2D receptor to which it binds is downregulated. Analyses of these genes in our Roswell Park cohort revealed that tumors with either KDM6A, SWI/SNF or both mutations had lower insignificant *z*-scores compared with tumors with neither mutation (Fig. 5e). The lower expression of NK cell ligands and receptors suggests that NK cells have reduced activity in bladder tumors with KDM6A and/or SWI/SNF mutations. Upregulation in any one of the NK cell ligands or receptor transcripts was associated with better overall and progression-free survival compared to tumors with no alterations in TCGA (Supplementary Figure 5A–B). Overall, these data indicate that activation of NK cells is one of the potential anti-tumor mechanisms in EZH2 inhibitor-treated HT1376 xenografts with KDM6A and SWI/SNF family member mutations.

### EZH2 inhibition-mediated NK cell activity is associated with reduced markers of pluripotency

**Fig. 6:**
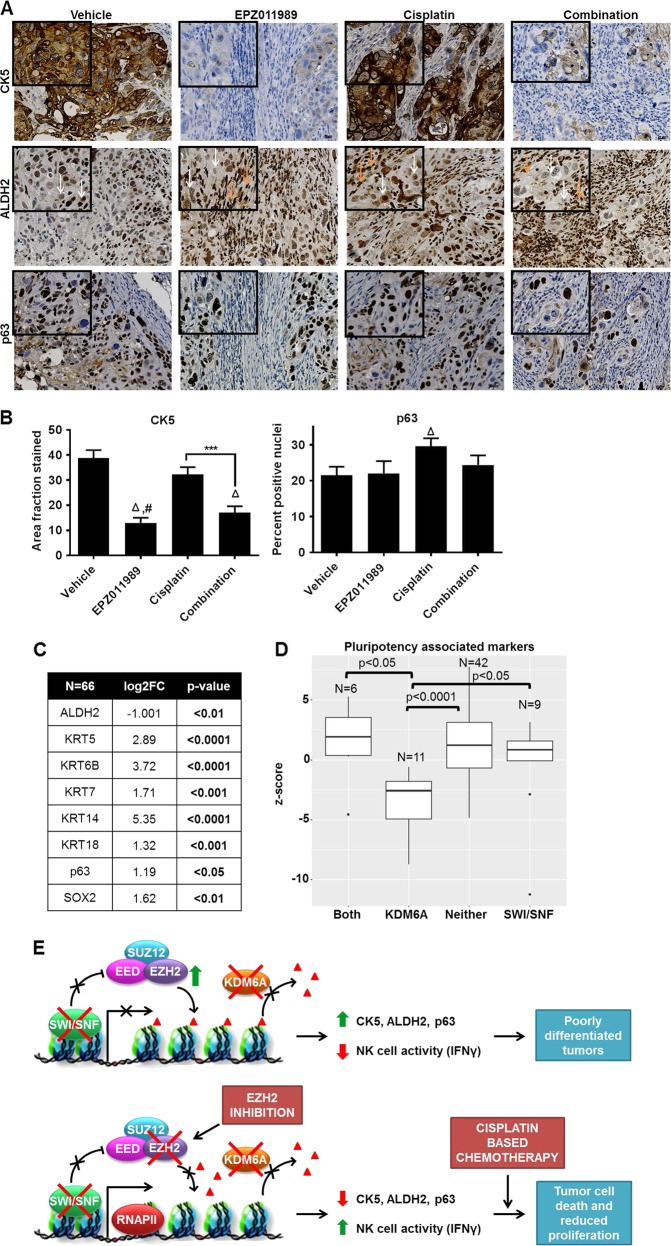
EZH2 inhibition-mediated NK cell activation reprograms bladder tumor cells by reducing expression of markers associated with pluripotent potential. **a**, **b** Markers of pluripotency in bladder cancer, CK5, ALDH2, and p63, were stained heterogeneously in all tumors. CK5 expression was significantly reduced and ALDH2 was reduced in EPZ011989 and combination-treated tumors compared with vehicle and cisplatin-treated tumors. p63 expression was significantly higher in cisplatin-treated tumors compared to vehicle group. Orange arrows indicate infiltrating cells and white arrows point to tumor cells. **c** RNA-sequencing analysis of Roswell Park muscle-invasive bladder tumors reveal significant upregulation of markers of cells with pluripotency potential, with the exception of ALDH2, compared to non-tumor tissues. **d** Pluripotency-associated markers were significantly lower in tumors with KDM6A mutation compared to all the mutation subtypes. **e** EZH2-mediated H3K27me3 deposition on histones is opposed by wild-type KDM6A and SWI/SNF complex. Tumor cells exhibit greater pluripotency potential and decreased NK cell activity when KDM6A and SWI/SNF loss of function mutations are unable to counteract EZH2 activity. EZH2 inhibition in this context increases NK cell activity measured by IFN-γ that mediates decrease in CK5 and ALDH2 that are pluripotency-associated markers. Greater NK cell activity and reduced pluripotency further augments cisplatin-based chemotherapy in muscle-invasive bladder cancer

EZH2 maintains stem-like features in cancer [38, 39], while cisplatin-based chemotherapy can enrich for stem-like cells leading to chemo-resistance [40, 41]. In addition, NK cell-mediated tumor differentiation can sensitize tumor cells to chemotherapy [36, 42]. Therefore, we tested the consequences of EPZ011989 and cisplatin treatment on pluripotency-associated markers both in vitro and in vivo. In vitro, we measured two markers: (1) CK5 that gives rise to muscle-invasive lesions in mice with BBN-induced bladder cancer [43], and (2) ALDH2 as an indicator of pluripotent potential [36]. Short-term EPZ011989 or siEZH2 treatment reduced CK5 expression in HT1376 and T24 cells and ALDH2 expression in T24 cells (Supplementary Figure 6A-D). CK5 was absent in UM-UC-3 cells, while HT1376 cells did not express ALDH2. ALDH2 expression remained unaltered in either EPZ011989 or siEZH2-treated UM-UC-3 cells (Supplementary Figure 6A–D). Cisplatin treatment increased ALDH2 expression in UM-UC-3 cells compared to vehicle-treated cells (Supplementary Figure 6E). In vivo, the expression of pluripotency markers CK5, ALDH2 and p63 were heterogeneous in all the xenografts that we assayed. Overall, CK5 staining was significantly lower in EPZ011989 and combination groups compared to vehicle control animals (Fig. 6a, b). A lower proportion of xenograft cells were positive for ALDH2 in EPZ011989 and combination groups (Fig. 6a). However, ALDH2 staining was more intense in infiltrating lymphocytes in EPZ011989 and combination-treated xenografts (Fig. 6a). Finally, p63 staining was significantly higher in cisplatin-treated tumors compared to vehicle group (Fig. 6a, b). To summarize, cisplatin treatment, downregulation of EZH2 protein by siEZH2 and pharmacological inhibition of EZH2 activity alter the expression of pluripotency markers in a subset of tested bladder cancer cells.

To determine whether the pluripotency markers are altered in bladder tumors and are associated with KDM6A and/or SWI/SNF tumors, we queried our Roswell Park genomics data. RNA transcripts that are associated with stem-like features were elevated in the Roswell Park cohort (Fig. 6c). In TCGA, either amplification or mRNA upregulation in any of these markers was associated with worse survival compared to patients with no alterations (Supplementary Figure 7A-B). Tumors with both KDM6A and SWI/SNF mutations had higher pluripotency *z*-scores compared to tumors with either single KMD6A, single SWI/SNF complex, or neither mutation (Fig. 6d). Higher pluripotency *z*-scores were associated with lower NK cell signature *z*-scores in tumors with both mutations compared to the other mutation subtypes. In conclusion, our results suggest that bladder cancer cells and xenografts with KDM6A and SWI/SNF family member mutations can benefit from EZH2 inhibition that increases NK cell activity leading to reduction in cells with pluripotency potential (Fig. 6e).

## Discussion

Our findings support that there is clinical utility in using EZH2 inhibitors for MIBC patients whose tumors have loss of function mutations in KDM6A and/or SWI/SNF complex. KDM6A and/or SWI/SNF family member mutations and EZH2 amplification/overexpression seem to be mutually exclusive in TCGA. Without KDM6A/SWI/SNF complex to oppose EZH2 function [8, 9, 19], EZH2 activity can be uninhibited to maintain a repressed transcriptional state without amplification or overexpression. These tumors may therefore present with inherently increased EZH2 activity that can be pharmacologically targeted by an EZH2 inhibitor. EZH2 inhibitor can also be beneficial in tumors with wild-type KDM6A and SWI/SNF complex where increased EZH2 activity occurs through post-translational modifications or other genetic alterations [44**–**46]. Post-translational modifications that enhance EZH2 activity include USP21-mediated de-ubiquitination [44] and JAK3-mediated phosphorylation [45, 46]. Genetic alterations that frequently occur in bladder tumors, for example, Rb1 loss and c-myc amplification, also drive EZH2 expression and activity [47, 48]. Therefore, patients who can potentially benefit from EZH2 inhibition would include those whose bladder tumors possess KDM6A and SWI/SNF mutations, Rb1/c-myc alterations and post-translational modifications of EZH2.

In our studies, EZH2 inhibition in HT1376 and T24 bladder cancer cells with KDM6A and/or SWI/SNF family member mutations increased levels of transcripts associated with NK cell activity. NK cells are important inhibitors of tumor development and progression [49, 50]. For example, TRAMP mice deficient in NKG2D/KLRK1, an NK cell receptor, develop more aggressive prostate cancer than their wild-type counterparts [51]. NK cell infiltration is capable of driving tumor cell lysis [37, 52], which is evident by greater TUNEL staining in EZH2 inhibitor-treated HT1376 xenografts. Enhanced NK cell infiltration in tumors can also drive differentiation of pluripotent cells to sensitize these cells to chemotherapy [36, 42].

Although the exact mechanism of epigenetic regulation of NK cell maturity and activity is not fully understood, EZH2 and KDM6A are known to regulate NK cell survival and activity in mice without any tumors [53, 54]. Non-tumor bearing mice with conditional EZH2 loss in hematopoietic stem and progenitor cells or those treated with EZH2 inhibitors show enhanced cell lineage commitment, survival and number of mature NK cells [54]. Pharmacological inhibition and knockdown of KDM6A reduces NK cell proliferation and IFN-γ production, a primary downstream NK cell target [53]. In our studies, *IFN-γ* transcript had the highest fold change in EZH2 inhibitor-treated HT1376 xenografts compared to vehicle-treated xenografts. IFN-γ has a crucial role in anti-tumor activity through immunological stimulation of macrophages and NK cells that drive tumor cell apoptosis [55]. Our data suggest that EZH2 inhibition in the context of KDM6A and/or SWI/SNF mutations increase NK cell activity that mediates IFN-γ release to cause tumor cell death. Epigenetic modifications other than histone methylation can also enhance NK cell-mediated tumor cell killing. AML cells pre-treated with Decitabine, a DNA hypomethylating agent, display increased susceptibility to NK cell-mediated killing in leukemic cell lines, xenografts and patient-derived cells [56]. DNA hypomethylation augments NK cell-mediated lysis by increasing NKG2D-ligand expression that allows recognition of tumor AML cells by NKG2D receptor (encoded by Killer Cell Lectin Like Receptor K1, *KLRK1*) on NK cells [57]. In our studies, EPZ011989 and combination treatments amplified transcript levels of genes including *CD86*, *MIP-1α*, *CD3D*, as well as CD56 and NCR1 protein levels that are indicative of active NK cells. This increase in NK cell activity is associated with greater tumor cell death as measured by TUNEL staining. Overall, these findings suggest to us that NK cell-mediated tumor cell killing may be partially response for anti-tumor activity observed with EZH2 inhibition alone and in combination with cisplatin in bladder cancer xenografts in nude mice.

Athymic nude mice have a limited immune cell composition that includes NK and B-cells but not T cells. It is possible that EZH2 inhibition in either immune-competent mice or human microenvironments, affects other components of the immune system in addition to NK cells. The cross talk between a fully developed immune system in an immune-competent host and its response to EZH2 inhibition in tumor cells remain to be fully elucidated. EZH2 inhibition in the HT1376 xenografts also increases mRNA of *PD-L1*. Others have shown that EZH2 inactivation can potentially synergize with checkpoint-based immunotherapy including anti-CTLA4 and PD-L1 in preclinical models of melanoma and ovarian cancer [58, 59]. It will be fascinating to study if EZH2 inhibition can augment PD-L1-based immunotherapy, an FDA approved drug for treating metastatic bladder cancer.

Cisplatin-based chemotherapy is the FDA-approved frontline treatment for MIBC. Therefore, it is likely that clinical testing of EZH2 inhibitors will involve patients that are pre-treated with cisplatin. We show that cisplatin raised H3K27me3 levels, an indication of increased EZH2 activity in vitro. Another study shows that chemo-resistant tumors from bladder cancer patients maintain or overexpress EZH2 protein levels after chemotherapy compared to patients who are chemo-sensitive [60]. Acquired chemo-resistance in cancer cells [40] is also associated with EZH2 overexpression that enriches for cells with stem cell-like features [61, 62]. Reducing the population of cells with pluripotent features can potentially augment chemotherapy [63, 64]. It has also been shown that NK cells can drive differentiation of tumor cells which augment cisplatin therapy [36, 42]. NK cells can also increase cytotoxicity of colon cancer stem-like-cells pre-treated with 5-fluorouracil or oxaliplatin-based chemotherapy [37]. These authors further investigated the efficacy of adoptive NK cell therapy in combination with chemotherapy in comparison to chemotherapy alone in colon cancer patients [37]. Although the numbers are small, patients treated with combination therapy had significantly higher overall and progression-free survival compared to the chemotherapy alone arm [37]. In our studies, EZH2 inhibition reduces expression of pluripotency markers including CK5, ALDH2, and p63. Based on these observations, our data suggest that EZH2 inhibition-mediated increase in NK cell activity has the potential to boost response to cisplatin therapy through increased cytotoxicity of pluripotent cells or tumor cell differentiation. This can occur independently of KDM6A and SWI/SNF complex mutations where prior cisplatin-based chemotherapy can mediate increased EZH2 activity. In MIBC where cisplatin and PD-1/PD-L1 are the only two FDA approved treatments, EZH2 inhibition represents an additional therapeutic option that needs to be fully explored.

## Supplementary information

Supplemental figures and tables
