# Supplementary material for: Inhibition of EZH2 induces NK cell-mediated differentiation and death in muscle-invasive bladder cancer
Source: Cell Death Differ. 2019 Jan 28;26(10):2100–14. doi: 10.1038/s41418-019-0278-9 (PMC6748105; doi:10.1038/s41418-019-0278-9)
Supplement: Supplementary file 1 — Supplemental figures and tables [file 41418_2019_278_MOESM1_ESM.pdf]

Supplementary Figure 1 Mutations and gene expression alterations in KDM6A and SWI/SNF complex affect overall and progression-free survival in muscle-invasive bladder cancer dataset in The Cancer Genome Atlas (TCGA).

(A). Oncoprint from TCGA (n=412) shows the percent of MIBC patients affected by mutations or mRNA alterations in EZH2, KDM6A and members of the SWI/SNF complex. **(B).** MIBC patients with loss of function mutations or mRNA downregulation in KMD6A or SWI/SNF complex genes present with worse overall survival and significantly reduced progression-free survival compared with patients with no alterations. EZH2 amplification and/or mRNA overexpression patients have shorter overall and progression-free survival compared to MIBC patients with no EZH2 alterations.

Supplementary Figure 2 EZH2 inhibition and cisplatin treatment alter H3K27me3 levels in bladder cancer cells.

(A). H3K27me3 levels are reduced in HT1376, T24 and UM-UC-3 cells treated with EPZ011989 for 4 days. Total H3 is used as a loading control. **(B).** H3K27me3 levels are increased with 48 hours of cisplatin treatment. Total H3 is used as a loading control.

Supplementary figure 3 EZH2 inhibitor reduces tumor proliferation *in vivo*.

(A). The treatment schedule did not affect the body weight of nude mice. The Y-axis represents body weight in grams, and the X-axis represents the number of treatment days. Error bars represent standard error of means of all the mice in each group. **(B)-(E).** Graphs represent tumor volumes of individual mice in each treatment group. The Y-axis represents tumor volume in $mm^{3,}$ and the X-axis represents the number of treatment days.

Supplementary Figure 4 RNA-sequencing of EZH2 treated cells and tumors revealed alteration of large number of gene transcripts.

(A)-(D). Venn diagrams show the number of transcripts that are altered in the short term EZH2 inhibitor studies *in vitro* and long-term xenograft studies *in vivo*.

Supplementary Figure 5 mRNA upregulation of at least one transcript associated with NK cell activation is associated with increased overall and progression-free survival in the TCGA MIBC cohort.

(A). Oncoprint from TCGA (n=412) shows the percent of MIBC patients affected by mutations or mRNA alterations that are related to NK cell activation. **(B).** MIBC patients with mRNA upregulation in any of the listed genes present with better overall and progression-free survival compared with patients with no alterations.

Supplementary Figure 6 Short-term EZH2 knockdown and cisplatin treatment alter markers of cells with pluripotent potential.

(A) HT1376, T24 and UM-UC-3 cells treated with EPZ011989 (1μM) for 4 days show reduced expression of ALDH2 and CK5 compared to vehicle treated cells. The numbers below the bands represent semi-quantitative analysis of band intensities using Image J. The numbers are normalized to tubulin (loading control) and to vehicle treatments. **(B)-(C).** EZH2 knockdown using short interference RNA reduces the levels of EZH2 as well as its target H3K27me3 levels. GAPDH and total Histone H3 are used as loading control. **(D).** EZH2 knockdown with short interference RNA reduces CK5 and ALDH2 levels. Tubulin is used as loading control. The numbers below the bands represent a semi-quantitative analysis of band intensities using Image J. The numbers are

normalized to tubulin (loading control) and vehicle treatments. **(E).** HT1376, T24 and UM-UC-3 cells treated with Cisplatin (0.25 μg/ml) for 48 hours showed increased ALDH2 and CK5 expression compared to vehicle-treated cells. The numbers below the bands represent a semi-quantitative analysis of band intensities using Image J. The numbers are normalized to tubulin (loading control) and vehicle treatments.

Supplementary Figure 7 mRNA upregulation of at least one transcript associated with pluripotency is associated with reduced overall and progression-free survival in the TCGA MIBC cohort

(A). Oncoprint from TCGA (n=412) shows the percent of MIBC patients affected by mutations or mRNA alterations in genes that are associated with pluripotency potential. **(B).** MIBC patients with mRNA upregulation in any of the listed genes present with worse overall and progression-free survival compared with patients with no alterations.

Supplementary Table 1 lists the types of mutations found in KDM6A and SWI/SNF complex members found in the RPCCC cohort

Supplementary Table 2 Mutations in HT1376, T24 and UM-UC-3 cells

Supplementary Table 3 p-values for *in vitro* experiments

Supplementary Table 4 p-values for *in vivo* experiments

Supplementary Table 5 EZH2 inhibition mediated upregulated transcripts single agent EPZ011989 and combination treated HT1376 xenografts *in vivo*

Supplementary Table 6 EZH2 inhibition mediated upregulated transcripts in HT1376 cells *in vitro*

Supplementary Table 7 EZH2 inhibition mediated upregulated transcripts in T24 cells *in vitro*

Supplementary Table 8 EZH2 inhibition mediated common upregulated transcripts in HT1376 and T24 cells *in vitro*

Supplementary Table 9 EZH2 inhibition mediated common upregulated transcripts in HT1376 *in vitro* and HT1376 xenografts *in vivo*

Supplementary Figure 1 Mutations and gene expression alterations in KDM6A and SWI/SNF complex affect overall and progression-free survival in muscle-invasive bladder cancer dataset in The Cancer Genome Atlas (TCGA)

(A)

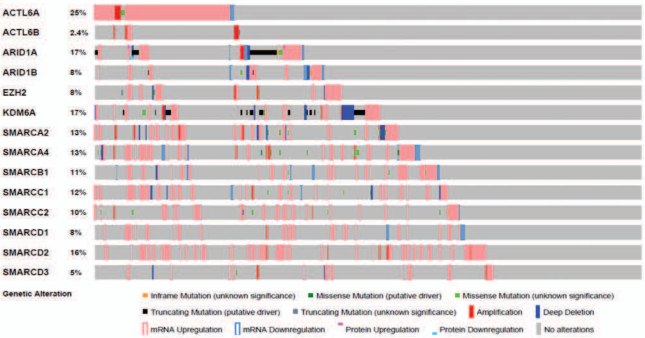

(B)

**Overall survival (p=0.363)**	Number of Cases, Total	Number of Cases, Deceased	Median Months Survival
Cases with at least one nonsense mutation or mRNA downregulation in selected genes (except EZH2)	36	16	30.91
Cases without any alterations	114	48	61.4
**Progression-free survival (p=0.034)**	Number of Cases, Total	Number of Cases, Relapsed/Progressed	Median Months Disease-free
Cases with at least one nonsense mutation or mRNA downregulation in selected genes (except EZH2)	30	18	17.35
Cases without any alterations	87	31	87.48
**Overall survival (p=0.868)**	Number of Cases, Total	Number of Cases, Deceased	Median Months Survival
Cases with EZH2 amplification or overexpression	**26**	**13**	**28.38**
Cases without any alterations	**379**	**167**	**33.11**
**Progression-free survival (p=0.428)**	Number of Cases, Total	Number of Cases, Relapsed/Progressed	Median Months Disease-free
Cases with EZH2 amplification or overexpression	**23**	**14**	**27.83**
Cases without any alterations	**295**	**128**	**32.59**

Supplementary Figure 2 EZH2 inhibition and cisplatin treatment alter H3K27me3 levels in bladder cancer cells

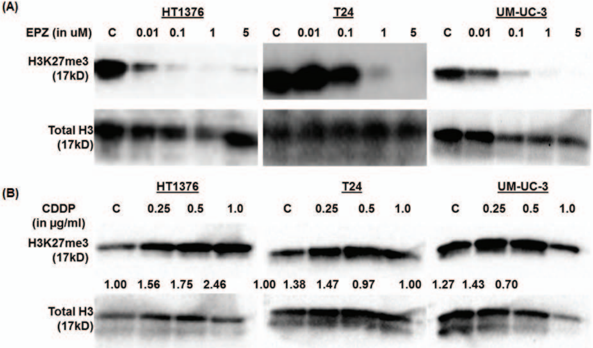

Supplementary figure 3 EZH2 inhibitor reduces tumor proliferation *in vivo*

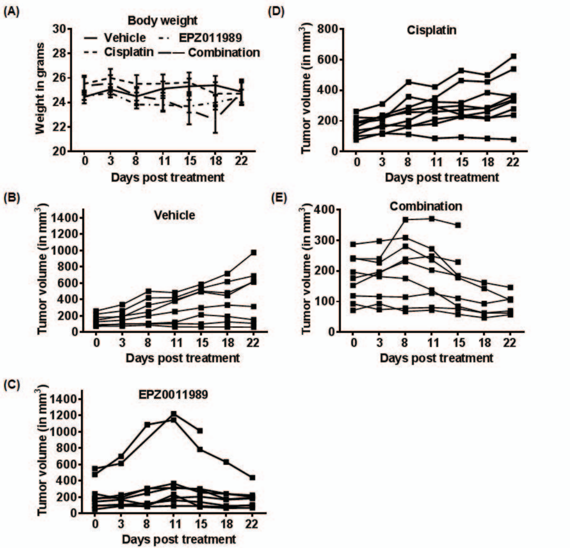

Supplementary Figure 4 RNA-sequencing of EZH2 treated cells and tumors revealed alteration of large number of gene transcripts

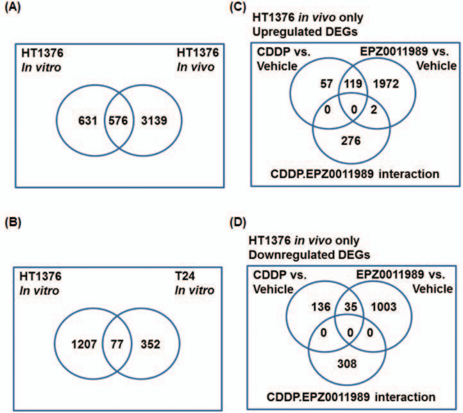

Supplementary Figure 5 mRNA upregulation of at least one transcript associated with NK cell activation is associated with increased overall and progression-free survival in the TCGA MIBC cohort

(A)

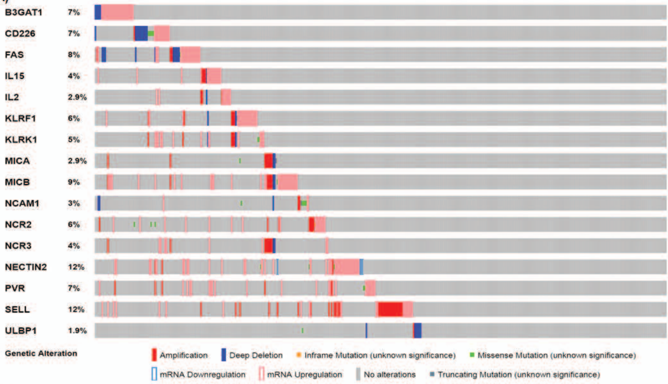

(B)

**Overall survival (p=0.477)**	Number of Cases, Total	Number of Cases, Deceased	Median Months Survival
Cases with at least one mRNA upregulation in selected genes	173	74	41.72
Cases without any alterations	176	82	32
**Progression-free survival (p=0.320)**	Number of Cases, Total	Number of Cases, Relapsed/Progressed	Median Months Disease-free
Cases with at least one mRNA upregulation in selected genes	136	58	37.78
Cases without any alterations	137	65	27.99

Supplementary Figure 6 Short term EZH2 knockdown and cisplatin treatment alters markers of cells with pluripotent potential

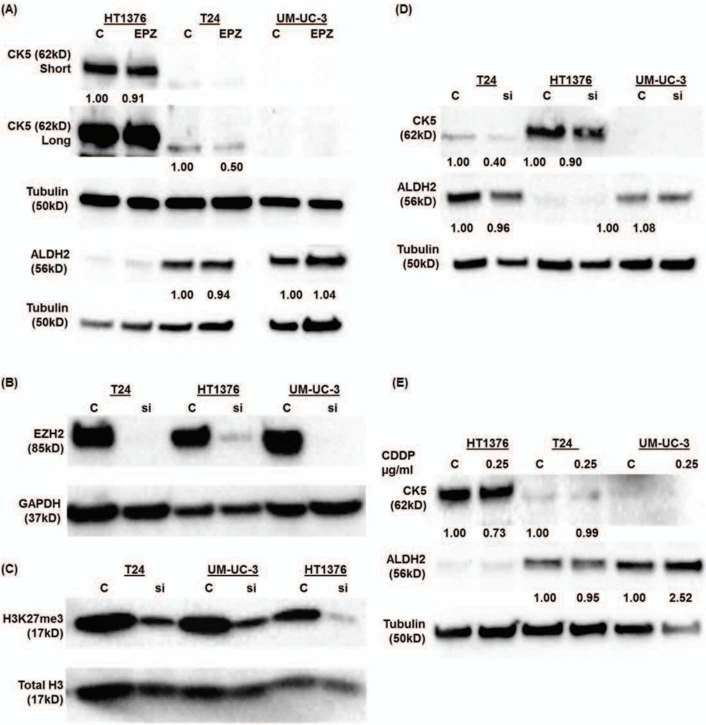

Supplementary Figure 7 mRNA upregulation of at least one transcript associated with pluripotency is associated with reduced overall and progression-free survival in the TCGA MIBC cohort

(A)

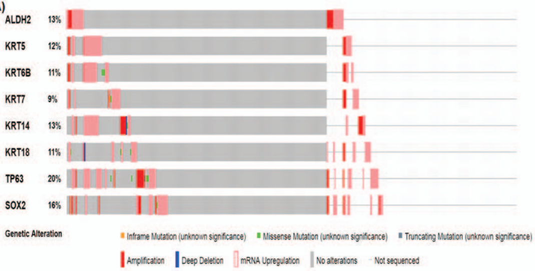

(B)

**Overall survival (p=0.744)**	Number of Cases, Total	Number of Cases, Deceased	Median Months Survival
Cases with at least one mRNA upregulation in selected genes	125	55	27.43
Cases without any alterations	268	116	34.95
**Progression-free survival (p=0.573)**	Number of Cases, Total	Number of Cases, Relapsed/Progressed	Median Months Disease-free
Cases with at least one mRNA upregulation in selected genes	100	46	27.83
Cases without any alterations	211	93	36.86

Supplementary table 1 lists the types of mutations found in KDM6A and SWI/SNF complex members found in the RPCCC cohort

Gene name	Mutation type	Amino acid change
**ACTL6B**	Missense	p.S200C
	Silent	p.G196G
**ARID1A**	Frame_Shift	p.109_112del, p.G922fs, p.728_734del, p.44_45del, p.G70fs, p.E2033fs, p.N833fs
	Missense	p.R1966H, p.E1806Q, p.S1001F, p.L1028V, p.P773L, p.P427L, p.E1643D, p.E1739Q, p.A1489D
	Nonsense	p.Q1493X, p.Q268X, p.Q585X, p.S350X, p.Q479X
	Silent	p.L117L, p.I1485I
**ARID1B**	In_Frame_Ins	p.A329delinsGA
	Nonsense	p.E1998X
**KDM6A**	Frame_Shift	p.1310_1313del, p.I1020fs, p.799_799del, p.N1150fs, p.A756fs, p.661_663del, p.S238fs, p.T521fs
	Missense	p.T548S, p.E363K, p.G137D, p.F984S, p.Q369R
	Nonsense	p.S884X, p.K831X, p.R519X, p.Q641X, p.Q677X, p.Q506X, p.R165X, p.Q555X, p.E946X, p.S706X, p.C1358X, p.K395X
	Silent	p.L547L, p.E1316E
	Splice Site	
**SMARCA4**	Intron	
	Missense	p.E798K
	Silent	p.P805P, p.L773L
**SMARCC1**	Missense	p.R955Q, p.E672Q
	Silent	p.P541P
**SMARCC2**	3'-UTR	

Supplementary table 2 Mutations in HT1376, T24 and UM-UC-3 cells

Gene name	HT1376	T24	UM-UC-3
ARID1A	c.557_570del14 (Deletion FS); c.3905C>G (Missense)		
SMARCA4	c.981A>C (Substitution, coding silent)		c.223-2A>G (Unknown)
SMARCD2	c.642C>G (Missense), c.666C>G (Missense)		
KDM6A	c.876-3C>G (Missense)	c.2683G>T, c.2704G>T (Nonsense)	c.3887T>G, c.3908T>G (Missense)

Supplementary Table 3 p-values for all *in vitro* experiments

**HT1376**							
	**Cell counts**		**Cell cycle (G1)**	**Cell cycle (S)**	**Cell cycle (G2/M)**		**Clonogenic assay**
**Treatment Comparison**	**p-value**						
Vehicle vs. Cisplatin	<0.05		<0.0001	0.56	<0.0001		<0.01
Vehicle vs. EPZ	<0.0001		<0.001	0.87	<0.01		<0.0001
Vehicle vs. Combination	<0.0001		<0.0001	0.05	<0.01		<0.0001
Cisplatin vs. EPZ	<0.01		0.03	0.95	0.10		<0.01
Cisplatin vs. Combination	<0.01		0.95	0.54	0.25		<0.0001
EPZ vs. Combination	0.97		<0.05	0.25	0.97		0.10
**T24**							
	**Cell counts**		**Cell cycle (G1)**	**Cell cycle (S)**	**Cell cycle (G2/M)**		**Clonogenic assay**
**Treatment Comparison**	**p-value**						
Vehicle vs. Cisplatin	<0.05		<0.0001	0.85	<0.0001		<0.0001
Vehicle vs. EPZ	<0.001		<0.001	0.75	0.75		0.14
Vehicle vs. Combination	<0.001		<0.0001	0.87	<0.0001		<0.0001
Cisplatin vs. EPZ	0.21		<0.0001	0.99	<0.0001		<0.05
Cisplatin vs. Combination	0.05		0.97	>0.99	0.96		0.59
EPZ vs. Combination	0.86		<0.0001	0.99	<0.0001		<0.05
**UM-UC-3**							
	**Cell counts**		**Cell cycle (G1)**	**Cell cycle (S)**	**Cell cycle (G2/M)**		**Clonogenic assay**
**Treatment Comparison**	**p-value**						
Vehicle vs. Cisplatin	0.20		<0.0001	<0.01	<0.0001		<0.0001
Vehicle vs. EPZ	<0.001		<0.05	<0.05	0.85		<0.05
Vehicle vs. Combination	<0.0001		<0.0001	0.93	<0.0001		<0.0001
Cisplatin vs. EPZ	<0.05		<0.001	0.92	<0.001		<0.01
Cisplatin vs. Combination	<0.05		<0.05	0.06	0.92		<0.05
EPZ vs. Combination	0.68		<0.0001	<0.05	<0.001		<0.001

Supplementary Table 4 p-values for *in vivo* xenograft experiment

Treatment Comparison	Tumor growth curve	End of study tumor volume	End of study tumor weight
Vehicle vs. CDDP	0.66	0.99	0.28
Vehicle vs. EPZ	0.13	0.25	0.23
Vehicle vs. EPZ+CDDP	<0.05	<0.05	<0.05
Cisplatin vs. EPZ	0.27	0.24	0.69
Cisplatin vs. EPZ+CDDP	0.05	<0.05	<0.05
EPZ vs. EPZ+CDDP	0.38	0.18	0.27

p-values for Ki67 level comparison	
**Treatment Comparison**	**p-value**
Vehicle vs. CDDP	<0.05
Vehicle vs. EPZ	<0.0001
Vehicle vs. EPZ+CDDP	0.7787
Cisplatin vs. EPZ	<0.0001
Cisplatin vs. EPZ+CDDP	0.1047
EPZ vs. EPZ+CDDP	<0.0001

p-values for for H3K27me3 level comparison	
**Treatment Comparison**	**p-value**
Vehicle vs. CDDP	0.91
Vehicle vs. EPZ	<0.01
Vehicle vs. EPZ+CDDP	<.001
Cisplatin vs. EPZ	<.001
Cisplatin vs. EPZ+CDDP	<.001
EPZ vs. EPZ+CDDP	0.72

p-values for for CD56 level comparison	
**Treatment Comparison**	**p-value**
Vehicle vs. CDDP	<0.05
Vehicle vs. EPZ	0.9038
Vehicle vs. EPZ+CDDP	<0.01
Cisplatin vs. EPZ	<0.05
Cisplatin vs. EPZ+CDDP	<0.05
EPZ vs. EPZ+CDDP	<0.0001

p-values for for NCR1 level comparison	
**Treatment Comparison**	**p-value**
Vehicle vs. CDDP	<0.01
Vehicle vs. EPZ	<0.01
Vehicle vs. EPZ+CDDP	<0.05
Cisplatin vs. EPZ	<0.001
Cisplatin vs. EPZ+CDDP	<0.01
EPZ vs. EPZ+CDDP	0.28

Supplementary table 5 EZH2 inhibition mediated upregulated transcripts single agent EPZ011989 and combination treated HT1376 xenografts *in vivo*

	Gene name	Log2FC
1	IFN-gamma	6.91
2	MHC class II	6.41
3	MIG	6.34
4	MIP-1-beta	5.86
5	CCL3L1	5.83
6	HLA-DQA1	5.54
7	G-protein beta/gamma	5.27
8	CD137(TNFRSF9)	5.25
9	MIP-1-alpha	5.15
10	PKC-theta	5.11
11	ICAM2	5.07
12	Collagen VI	5.05
13	CD3	4.99
14	TFPI-2	4.94
15	HLA-DPB1	4.92
16	MHC class II beta chain	4.92
17	HLA-DRB1	4.90
18	Amphiregulin	4.88
19	HLA-DPA1	4.85
20	CCL22	4.75
21	CD74	4.73
22	CEACAM1	4.72
23	ITK	4.58
24	Galpha(q)-specific peptide GPCRs	4.56
25	CD21	4.54
26	IL-7 receptor	4.51
27	IL7RA	4.51
28	G-protein alpha-i family	4.46
29	HLA-DQB1	4.46
30	HLA-DRA1	4.45
31	CSF1	4.38
32	PECAM1	4.37
33	alpha-M/beta-2 integrin	4.27
34	ITGB2	4.27
35	IL-2R gamma chain	4.23
36	IL-15 receptor	4.23
37	IL-2 receptor	4.23
38	IL-21 receptor	4.23
39	IL-21 receptor	4.23
40	HLA-DMB	4.08
41	CCL5	3.96

42	ENA-78	3.89
43	GCP2	3.89
44	GRO-1	3.87
45	IP10	3.87
46	HLA-DOA	3.87
47	G-CSF	3.82
48	IL-5	3.81
49	IL-24	3.74
50	CD244	3.69
51	NKG2A	3.69
52	IL-31RA	3.62
53	IL-35	3.52
54	IL-12 alpha	3.52
55	IL-2R alpha chain	3.51
56	IL23A	3.49
57	HLA-DMA	3.49
58	CD33	3.44
59	TNF-beta	3.35
60	SHPS-1	3.30
61	IL-12RB1	3.28
62	Cathepsin S	3.25
63	I-TAC	3.25
64	LAIR1	3.24
65	VCAM1	3.18
66	CFTR	3.14
67	IL-6	3.14
68	CD5	3.13
69	C1s	3.10
70	CD3 epsilon	3.06
71	PTX3	3.05
72	EBI3	3.05
73	ROR-alpha	2.98
74	IL-20	2.93
75	Tubulin alpha	2.91
76	HLA-F	2.85
77	PI3K reg class IB (p101)	2.85
78	ICAM1	2.82
79	HLA-B	2.79
80	IGF-1	2.79
81	MMP-13	2.77
82	LIF	2.76
83	TGF-beta	2.75
84	IL-11	2.71
85	PLC-gamma	2.71
86	PKC-alpha	2.70
87	CIITA	2.65
88	SDF-1	2.59

89	Alpha-actinin	2.58
90	CD86	2.57
91	CD86	2.57
92	ESAM	2.57
93	TR2(TNFRSF14)	2.49
94	MMP-9	2.47
95	ITGAL	2.46
96	DDR2	2.44
97	FCGR3A	2.41
98	PSMB9	2.39
99	Rac2	2.39
100	MASP1	2.38
101	C1r	2.36
102	GM-CSF	2.35
103	TNF-alpha	2.31
104	HLA-C	2.29
105	SOCS2	2.22
106	CCR4	2.21
107	L-selectin	2.21
108	BAFF(TNFSF13B)	2.21
109	IL-15RA	2.19
110	PLAUR (uPAR)	2.17
111	TNF-R2	2.17
112	TL1A(TNFSF15)	2.16
113	CCL17	2.13
114	C1qRp	2.08
115	HLA-H	2.08
116	DPP4	2.06
117	CX3CL1	2.04
118	C3	2.03
119	C5 convertase (C3b2Bb)	2.03
120	iC3b	2.03
121	HLA-A	2.01
122	SERPINE2	2.00
123	TAP1 (PSF1)	2.00
124	RANK(TNFRSF11A)	1.98
125	CD70(TNFSF7)	1.95
126	CD70(TNFSF7)	1.95
127	C5L2	1.95
128	PD-L1	1.94
129	MAGI-1(BAIAP1)	1.92
130	PD-L2	1.92
131	IL-7	1.88
132	IL-2R beta chain	1.87
133	PAI2	1.86
134	C1 inhibitor	1.85
135	PI3K cat class IB (p110-gamma)	1.84

136	SOCS3	1.84
137	STAT5	1.83
138	STAT5A	1.83
139	Serglycin	1.80
140	ITGAX	1.79
141	PAK1	1.79
142	CD137 ligand(TNFSF9)	1.77
143	DDR1	1.77
144	Galectin-9	1.75
145	CCL20	1.72
146	CCL20	1.72
147	Syk	1.71
148	CXCL16	1.71
149	IL-8	1.70
150	Talin	1.70
151	Factor B	1.67
152	ICOS-L	1.66
153	C4	1.63
154	NF-AT1(NFATC2)	1.60
155	PSMD3	1.59
156	ITGAM	1.58
157	LIGHT(TNFSF14)	1.58
158	ULBP2	1.54

Supplementary table 6 EZH2 inhibition mediated upregulated transcripts in HT1376 cells *in vitro*

	Gene name	Log2FC
1	PRKCI	5.47
2	Cyclin D2	5.42
3	CD30(TNFRSF8)	5.03
4	PECAM1	4.78
5	G-protein alpha-i family	4.41
6	MHC class II	4.41
7	G-protein beta/gamma	3.49
8	IL20RA	3.11
9	GRO-1	3.10
10	GRO-2	3.10
11	cPKC (conventional)	2.98
12	PKC-alpha	2.98
13	TIM-1	2.96
14	EBI3	2.91
15	MMP-13	2.89
16	Ephrin-B receptor 1	2.89
17	Stromelysin-1	2.73
18	TIM-3	2.68
19	TLR2	2.65
20	Nectin-3	2.64
21	TRAF5	2.59
22	PIP5KI	2.59
23	IP10	2.58
24	IL7RA	2.58
25	CCL3L1	2.53
26	PLC-gamma	2.49
27	PLC-gamma 2	2.49
28	PI3K cat class IB (p110-gamma)	2.48
29	Collagen V	2.39
30	MMP-9	2.36
31	CD137(TNFRSF9)	2.36
32	CD137 ligand(TNFSF9)	2.10
33	Histamine H1 receptor	2.10
34	CaMK II beta	2.10
35	Tubulin (in microtubules)	1.95
36	Tubulin beta	1.95
37	PI3K reg class IA	1.89
38	Rac2	1.85
39	Galpha(q)-specific peptide GPCRs	1.84
40	ARAP3	1.82

41	IL-8	1.68
42	Tubulin alpha	1.67
43	TR2(TNFRSF14)	1.64
44	CD70(TNFSF7)	1.61
45	IP3 receptor	1.60
46	ICOS-L	1.55
47	ENA-78	1.49
48	GCP2	1.49
49	Talin	1.48
50	TLN2	1.48
51	Actin	1.46
52	CCL5	1.38
53	SLAP-130(ADAP)	1.36
54	IL-24	1.33
55	NF-AT2(NFATC1)	1.28
56	IL27RA	1.23
57	MLCK	1.20
58	MYLK1	1.20

Supplementary table 7 EZH2 inhibition mediated upregulated transcripts in T24 cells *in vitro*

	**Gene name**	**Log2FC**
1	GUCY1A2	2.15
2	PKA-reg (cAMP-dependent)	1.94
3	NF-AT	1.52
4	PLC-beta	1.47
5	p67-phox	1.29

Supplementary table 8 EZH2 inhibition mediated common upregulated transcripts in HT1376 and T24 cells *in vitro*

	Gene name	HT log2FC	T24 log2FC
1	NF-AT1(NFATC2)	4.75	2.35
2	MMP-1	3.57	2.65
3	Thy-1	2.93	2.26
4	I-TAC	2.51	2.36
5	G-protein alpha-i family	2.09	2.16
6	CXCR4	2.00	1.25
7	BFL1	1.98	2.14
8	P2Y1	1.59	1.47

Supplementary table 9 EZH2 inhibition mediated common upregulated transcripts in HT1376 *in vitro* and HT1376 xenografts *in vivo*

1	ASK1 (MAP3K5)
2	BFL1
3	CalDAG-GEFII
4	CARD11
5	CCL3L1
6	CCL5
7	CD137 ligand(TNFSF9)
10	CD137(TNFRSF9)
11	CD30(TNFRSF8)
12	CD70(TNFSF7)
13	EBI3
14	ENA-78
15	Galpha(q)-specific peptide GPCRs
16	GCP2
17	G-protein alpha-i family
18	G-protein beta/gamma
19	GRO-1
20	GRO-2
21	ICOS-L
23	IL-24
24	IL27RA
25	IL7RA
26	IL-8
27	IP10
28	I-TAC
29	MHC class II
31	MICB
32	NF-AT
33	NF-AT1(NFATC2)
34	PI3K cat class IA
36	PI3K cat class IA (p110-delta)
37	PI3K cat class IB (p110-gamma)
41	PKC-alpha
42	PKC-theta
43	PKC-theta
44	PLC-gamma
46	Rac2
47	RANK(TNFRSF11A)
48	SLAP-130(ADAP)
50	Talin
51	TNF-alpha

53	TR2(TNFRSF14)
54	TRAF1
55	Tubulin alpha
